# Supplementary material for: A Humanized Yeast Phenomic Model of Deoxycytidine Kinase to Predict Genetic Buffering of Nucleoside Analog Cytotoxicity
Source: Genes (Basel). 2019 Sep 30;10(10):770. doi: 10.3390/genes10100770 (PMC6826991; doi:10.3390/genes10100770)
Supplement: Supplementary file 1 [file genes-10-00770-s001.zip › Additional_Files/Additional_File7_GOTermHeatmaps.zip!/Additional_File7_GOTermHeatmaps/DNA_biosynthetic_process.pdf]

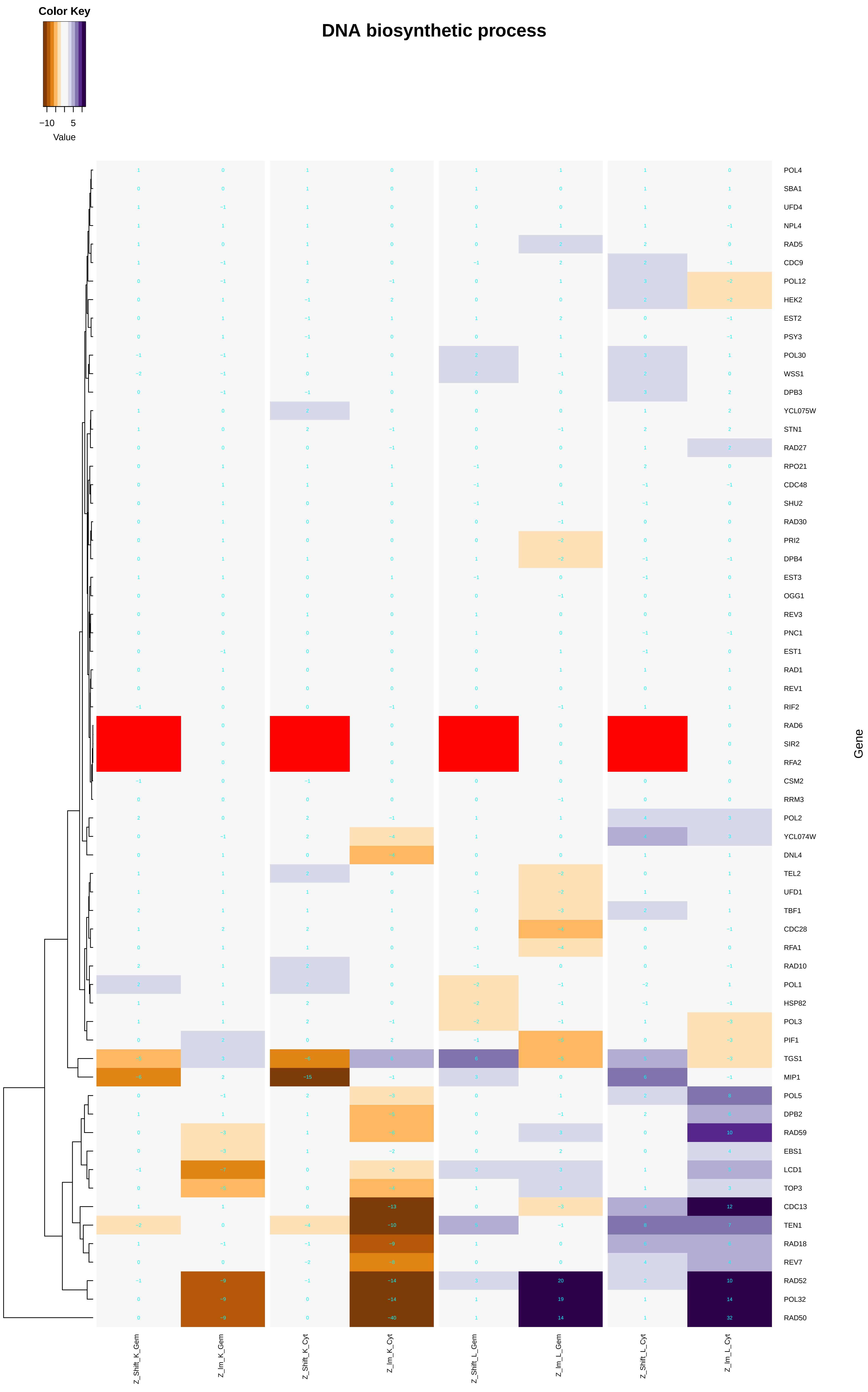
DNA biosynthetic process
Color Key
−10
5
Value
POL4
SBA1
UFD4
NPL4
RAD5
CDC9
POL12
HEK2
EST2
PSY3
POL30
WSS1
DPB3
YCL075W
STN1
RAD27
RPO21
CDC48
SHU2
RAD30
PRI2
DPB4
EST3
OGG1
REV3
PNC1
EST1
RAD1
REV1
RIF2
RAD6
SIR2
RFA2
CSM2
RRM3
POL2
YCL074W
DNL4
TEL2
UFD1
TBF1
CDC28
RFA1
RAD10
POL1
HSP82
POL3
PIF1
TGS1
MIP1
POL5
DPB2
RAD59
EBS1
LCD1
TOP3
CDC13
TEN1
RAD18
REV7
RAD52
POL32
RAD50
Gene
Z_Shift_K_Gem
Z_Im_K_Gem
Z_Shift_K_Cyt
Z_Im_K_Cyt
Z_Shift_L_Gem
Z_Im_L_Gem
Z_Shift_L_Cyt
Z_Im_L_Cyt

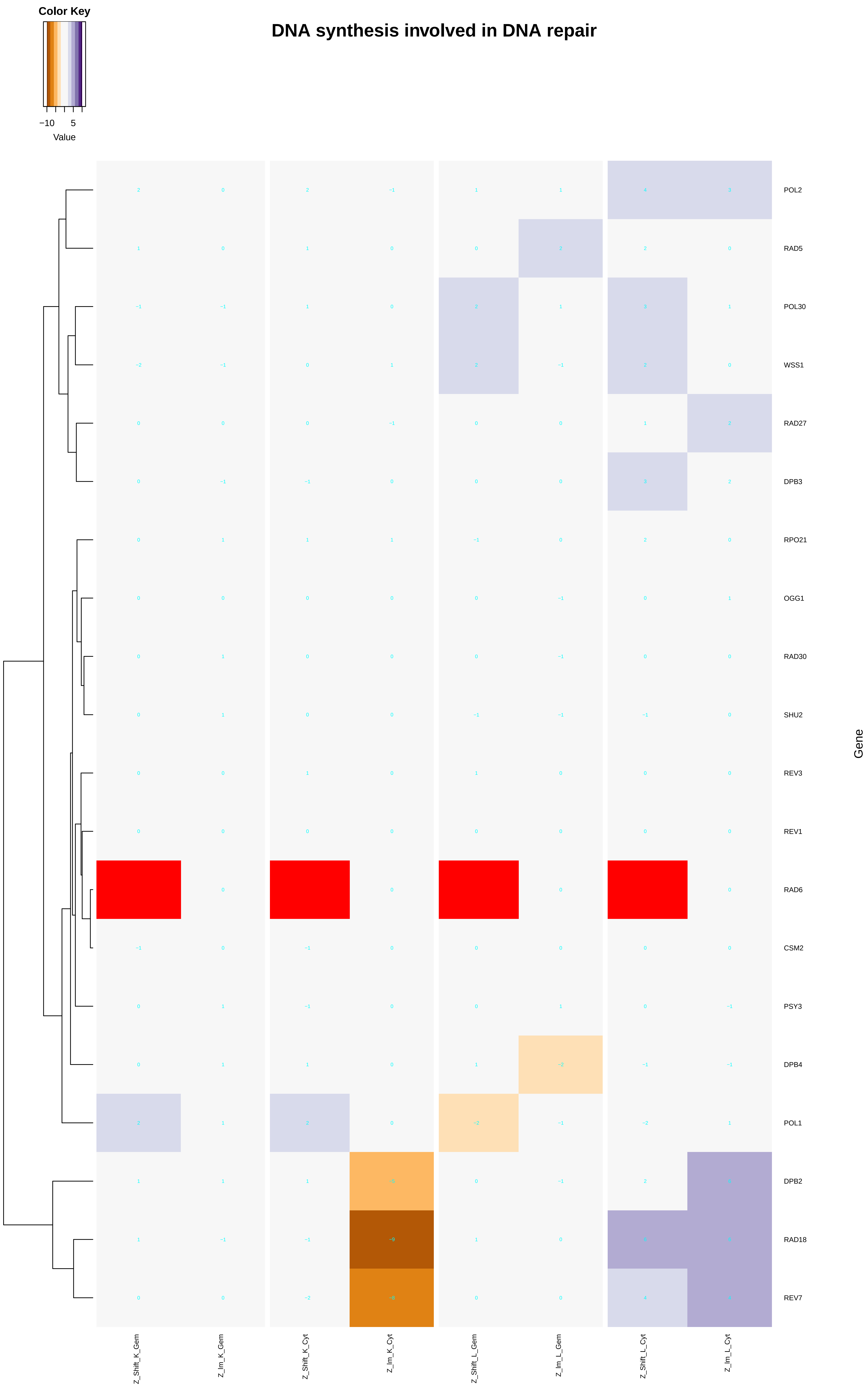
DNA synthesis involved in DNA repair
Color Key
−10
5
Value
POL2
RAD5
POL30
WSS1
RAD27
DPB3
RPO21
OGG1
RAD30
SHU2
REV3
REV1
RAD6
CSM2
PSY3
DPB4
POL1
DPB2
RAD18
REV7
Gene
Z_Shift_K_Gem
Z_Im_K_Gem
Z_Shift_K_Cyt
Z_Im_K_Cyt
Z_Shift_L_Gem
Z_Im_L_Gem
Z_Shift_L_Cyt
Z_Im_L_Cyt

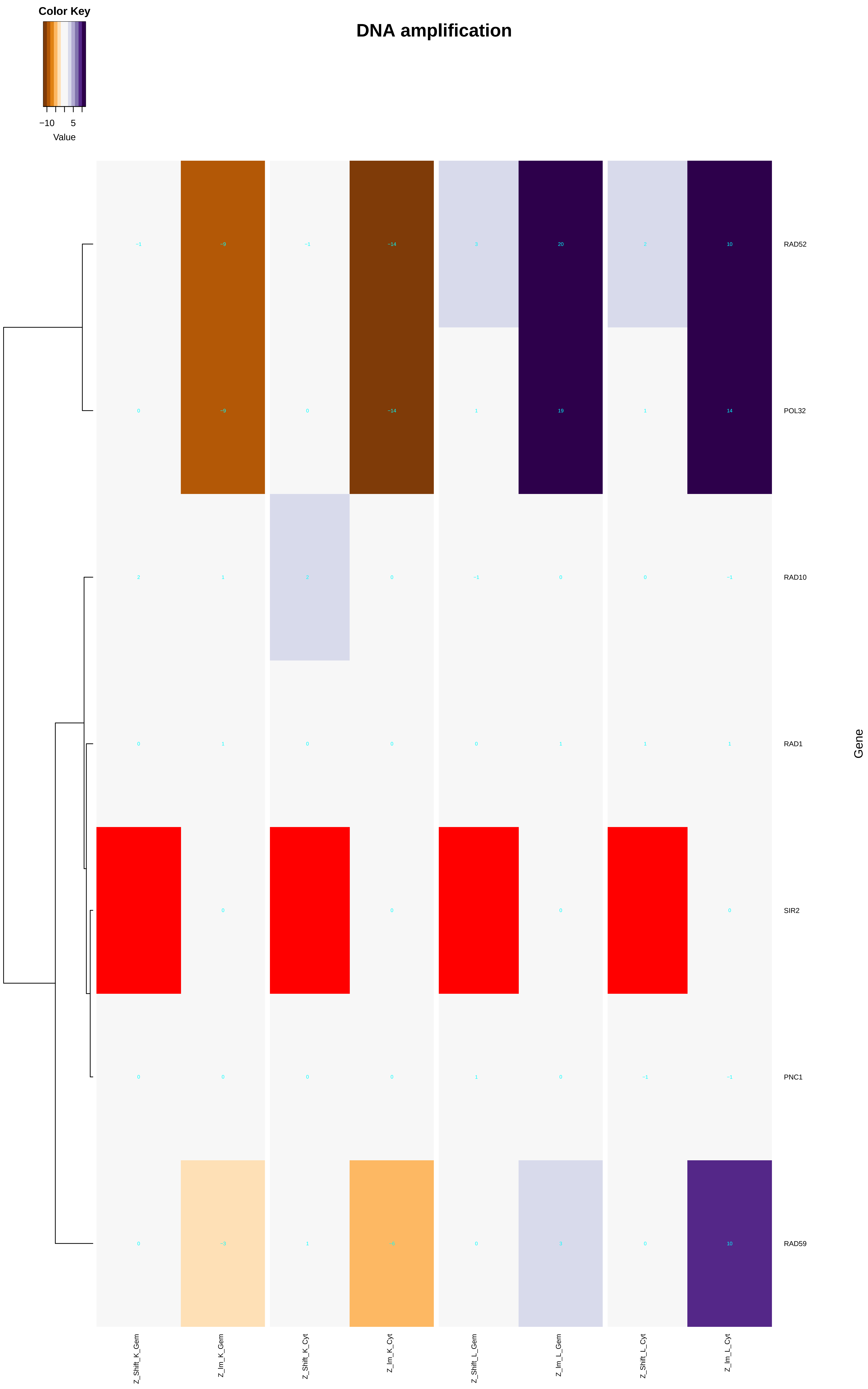
Color Key
−10
5
Value
DNA amplification
RAD52
POL32
RAD10
RAD1
SIR2
PNC1
RAD59
Gene
Z_Shift_K_Gem
Z_Im_K_Gem
Z_Shift_K_Cyt
Z_Im_K_Cyt
Z_Shift_L_Gem
Z_Im_L_Gem
Z_Shift_L_Cyt
Z_Im_L_Cyt

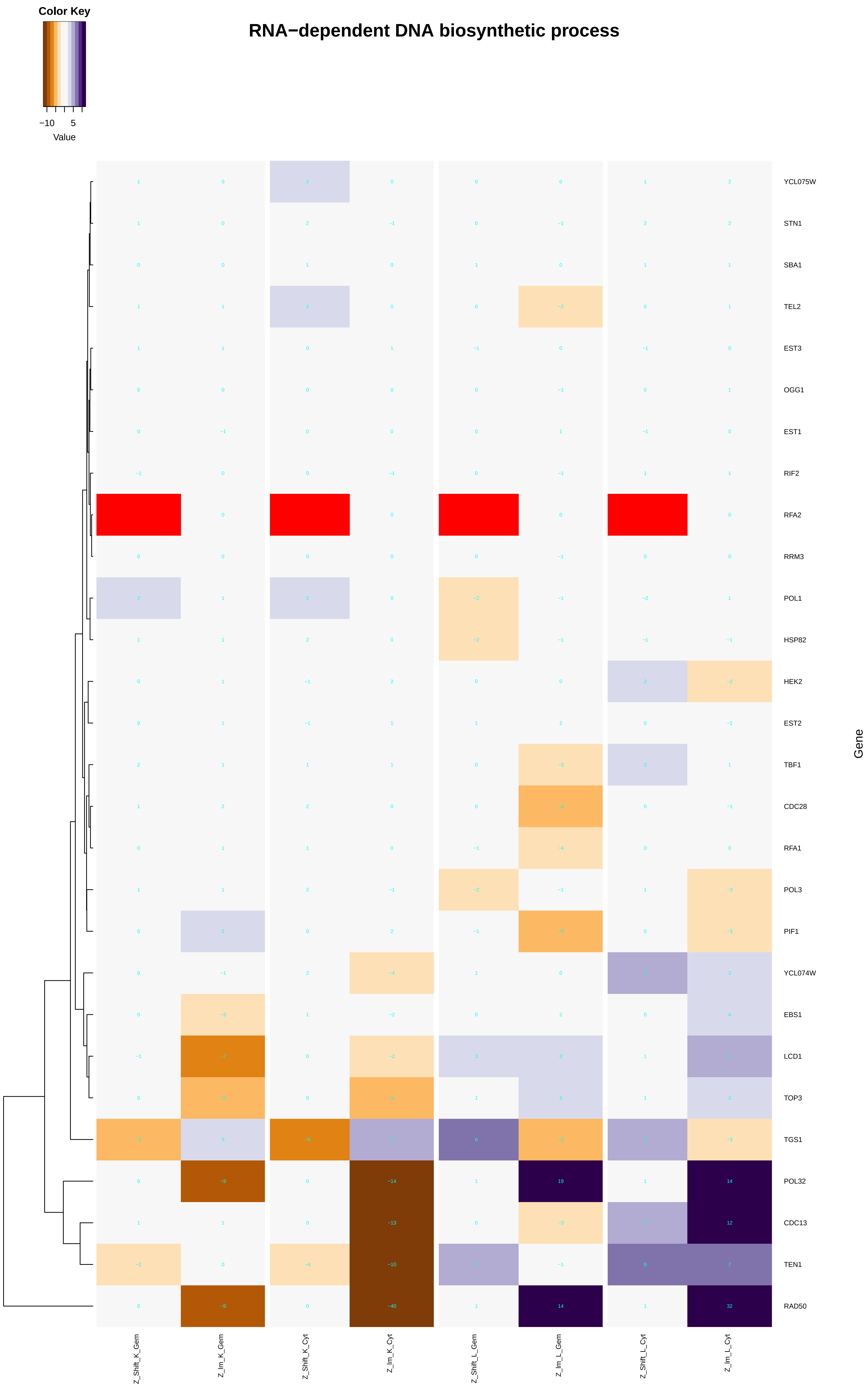
Color Key
−10 5
Value
RNA−dependent DNA biosynthetic process
YCL075W
STN1
SBA1
TEL2
EST3
OGG1
EST1
RIF2
RFA2
RRM3
POL1
HSP82
HEK2
EST2
TBF1
CDC28
RFA1
POL3
PIF1
YCL074W
EBS1
LCD1
TOP3
TGS1
POL32
CDC13
TEN1
RAD50
Gene
Z_Shift_K_Gem
Z_Im_K_Gem
Z_Shift_K_Cyt
Z_Im_K_Cyt
Z_Shift_L_Gem
Z_Im_L_Gem
Z_Shift_L_Cyt
Z_Im_L_Cyt

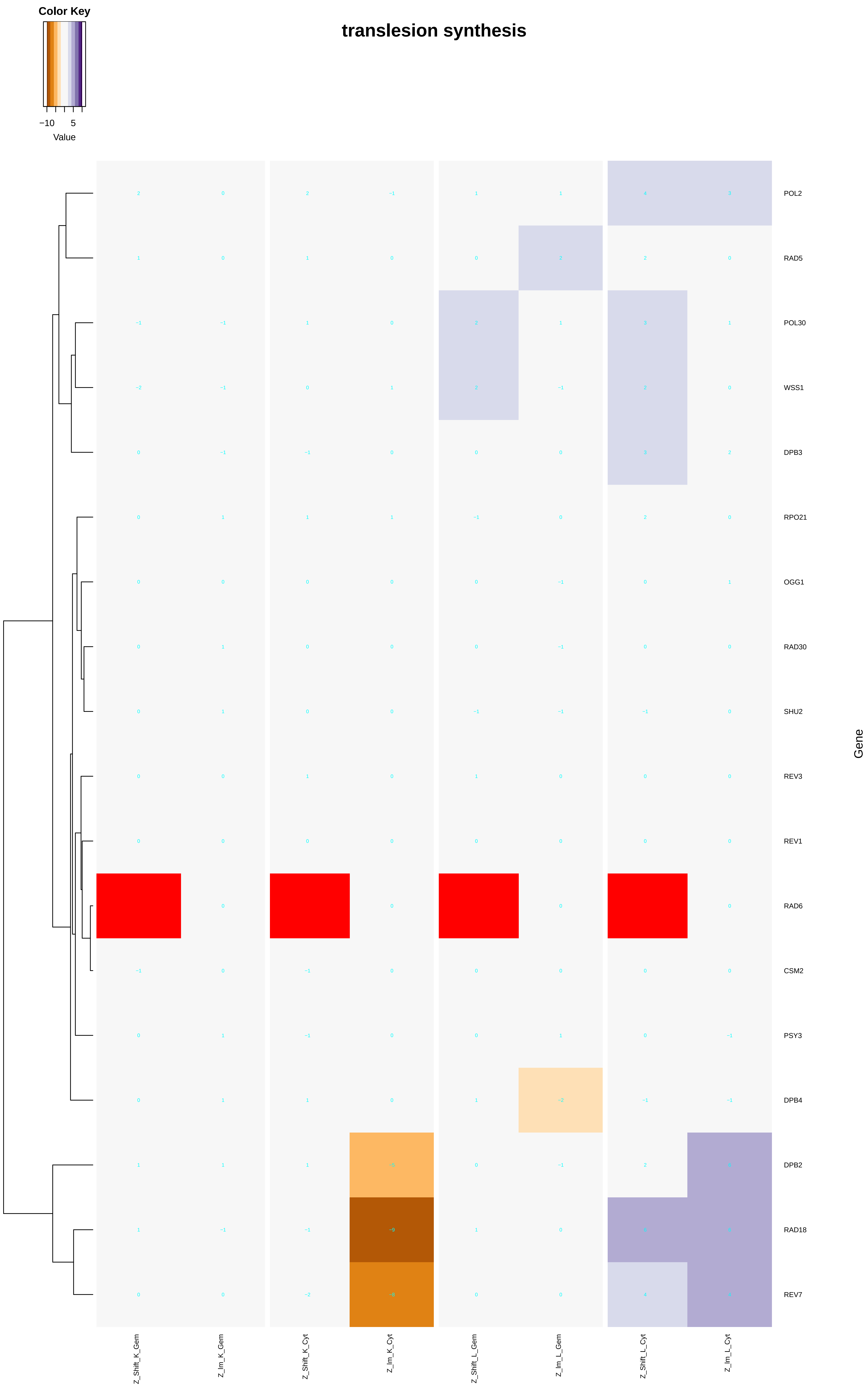

Color Key
−10
5
Value
translesion synthesis
POL2
RAD5
POL30
WSS1
DPB3
RPO21
OGG1
RAD30
SHU2
REV3
REV1
RAD6
CSM2
PSY3
DPB4
DPB2
RAD18
REV7
Gene
Z_Shift_K_Gem
Z_Im_K_Gem
Z_Shift_K_Cyt
Z_Im_K_Cyt
Z_Shift_L_Gem
Z_Im_L_Gem
Z_Shift_L_Cyt
Z_Im_L_Cyt

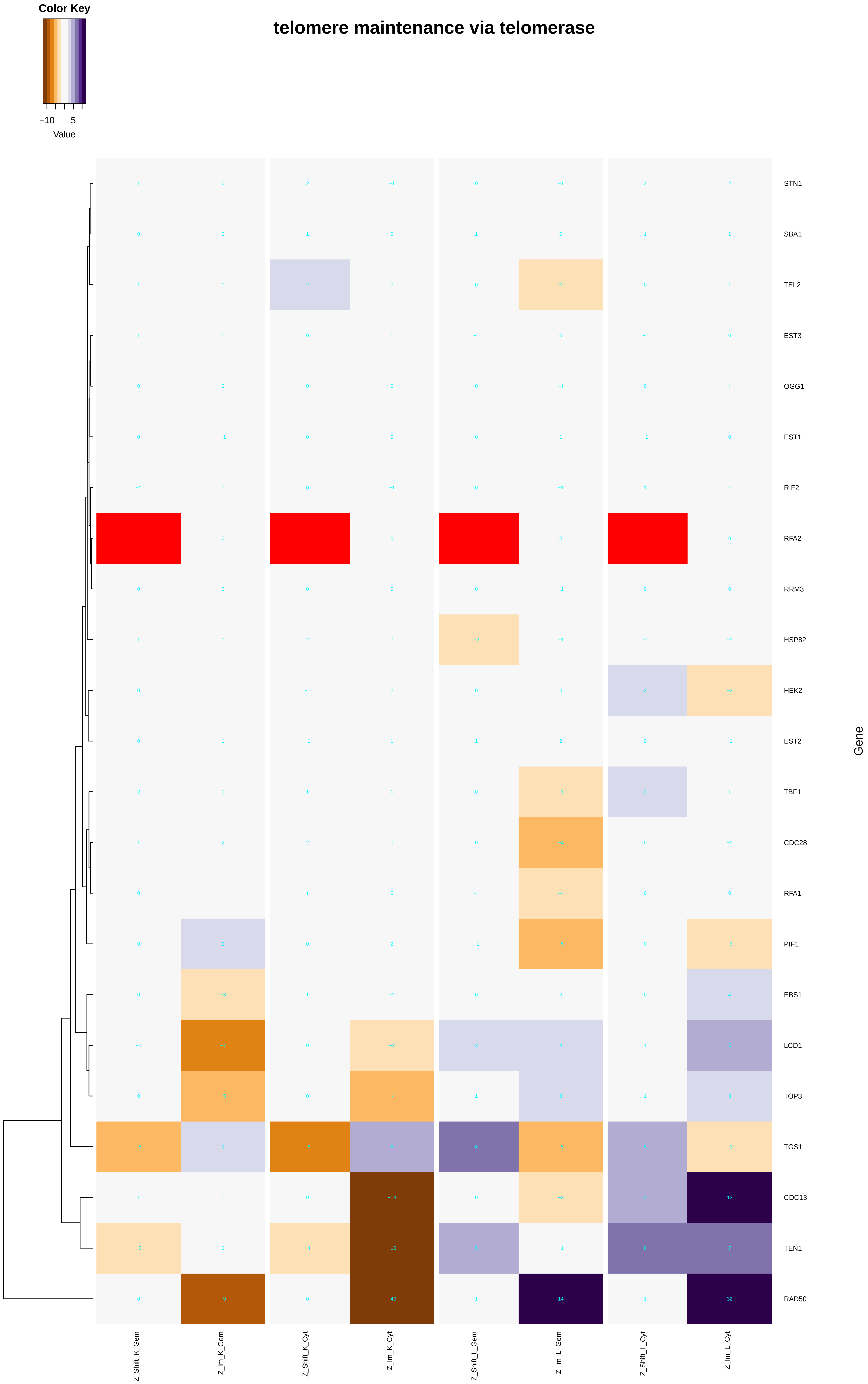

Color Key
−10 5
Value
telomere maintenance via telomerase
STN1
SBA1
TEL2
EST3
OGG1
EST1
RIF2
RFA2
RRM3
HSP82
HEK2
EST2
TBF1
CDC28
RFA1
PIF1
EBS1
LCD1
TOP3
TGS1
CDC13
TEN1
RAD50
Gene
Z_Shift_K_Gem
Z_Im_K_Gem
Z_Shift_K_Cyt
Z_Im_K_Cyt
Z_Shift_L_Gem
Z_Im_L_Gem
Z_Shift_L_Cyt
Z_Im_L_Cyt

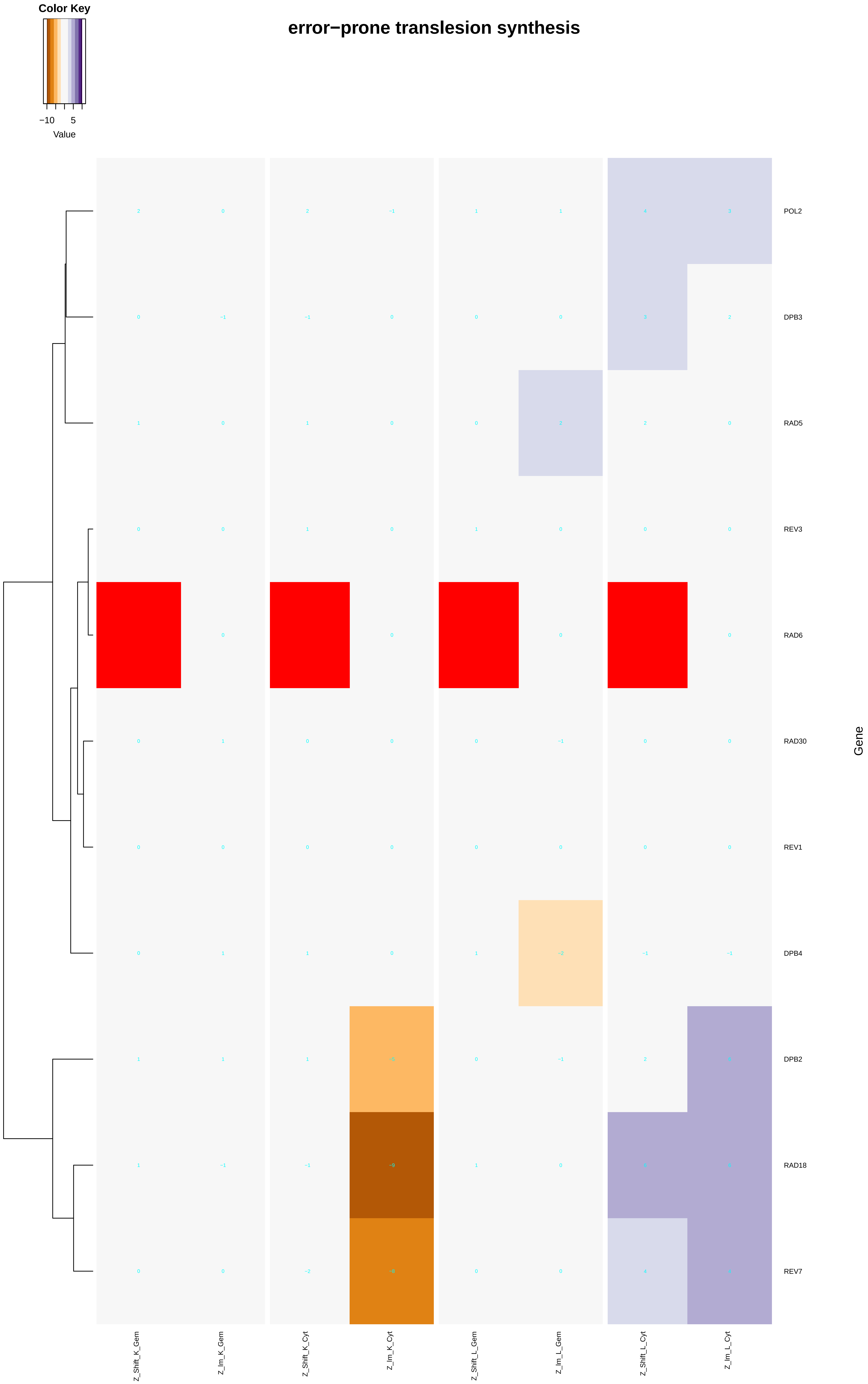
error−prone translesion synthesis
Color Key
−10
5
Value
POL2
DPB3
RAD5
REV3
RAD6
RAD30
REV1
DPB4
DPB2
RAD18
REV7
Gene
Z_Shift_K_Gem
Z_Im_K_Gem
Z_Shift_K_Cyt
Z_Im_K_Cyt
Z_Shift_L_Gem
Z_Im_L_Gem
Z_Shift_L_Cyt
Z_Im_L_Cyt

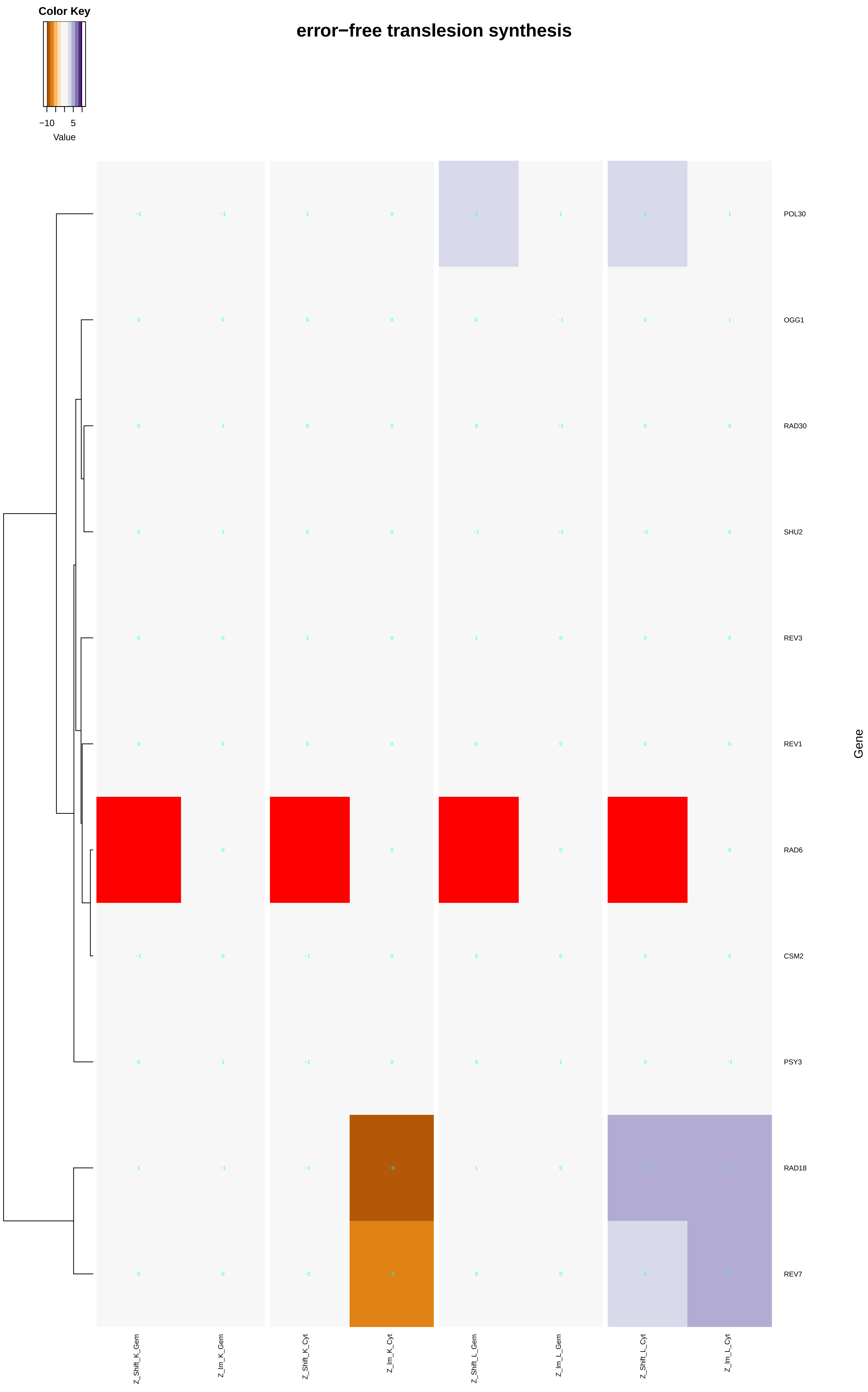
Color Key
−10
5
Value
error−free translesion synthesis
POL30
OGG1
RAD30
SHU2
REV3
REV1
RAD6
CSM2
PSY3
RAD18
REV7
Gene
Z_Shift_K_Gem
Z_Im_K_Gem
Z_Shift_K_Cyt
Z_Im_K_Cyt
Z_Shift_L_Gem
Z_Im_L_Gem
Z_Shift_L_Cyt
Z_Im_L_Cyt
